# The Effect of Balancing Selection on Population Differentiation: A Study with HLA Genes

**DOI:** 10.1534/g3.118.200367

**Published:** 2018-06-27

**Authors:** Débora Y. C. Brandt, Jônatas César, Jérôme Goudet, Diogo Meyer

**Affiliations:** * Departamento de Genética e Biologia Evolutiva, Universidade de São Paulo, São Paulo, SP, Brazil; † Department of Ecology and Evolution, University of Lausanne, Lausanne, Switzerland; ‡ Swiss Institute of Bioinformatics, University of Lausanne, Lausanne, Switzerland

**Keywords:** HLA, population differentiation, balancing selection

## Abstract

Balancing selection is defined as a class of selective regimes that maintain polymorphism above what is expected under neutrality. Theory predicts that balancing selection reduces population differentiation, as measured by F${}_{\text{ST}}$. However, balancing selection regimes in which different sets of alleles are maintained in different populations could increase population differentiation. To tackle the connection between balancing selection and population differentiation, we investigated population differentiation at the HLA genes, which constitute the most striking example of balancing selection in humans. We found that population differentiation of single nucleotide polymorphisms (SNPs) at the HLA genes is on average lower than that of SNPs in other genomic regions. We show that these results require using a computation that accounts for the dependence of F${}_{\text{ST}}$ on allele frequencies. However, in pairs of closely related populations, where genome-wide differentiation is low, differentiation at HLA is higher than in other genomic regions. Such increased population differentiation at HLA genes for recently diverged population pairs was reproduced in simulations of overdominant selection, as long as the fitness of the homozygotes differs between the diverging populations. The results give insight into a possible “divergent overdominance” mechanism for the nature of balancing selection on HLA genes across human populations.

Natural selection is one of the forces shaping the genetic variation within and the differentiation between populations. In the case of a locus where a variant is favored in one population but not in another (*i.e.*, in which selection drives local adaptation), we expect differentiation to exceed that under purely demographic processes (Lewontin and Krakauer 1973). This is the case for well known examples, such as the regulatory variant that generates lactase persistence in adulthood, which is more frequent in Europeans (Bersaglieri *et al.* 2004) and variants of the *EPAS1* gene that provide adaptation to high altitude in Tibetans (Xu *et al.* 2011). Purifying selection, on the other hand, is more common and removes most variants which would contribute to differences among populations. Therefore, it is expected to reduce genetic differentiation at the focal locus with respect to a strictly neutral scenario (*e.g.*, Barreiro *et al.* (2008)), while differentiation in surrounding genomic regions may increase due to the lower effective population size (Charlesworth *et al.* 1997).

A third regime, balancing selection, is related to diversity and differentiation in more complex ways. By definition balancing selection encompasses all selective regimes that result in increased genetic diversity relative to neutral expectations. The increased variability can result from a variety of processes, often with different underlying biological properties: frequency-dependent selection, heterozygote advantage, selection varying over temporal and geographic scales (Andrés 2011). As a consequence, the expectations regarding population differentiation under balancing selection represent a challenging theoretical and empirical question.

Across human populations, the loci with the strongest evidence for balancing selection are the classical HLA class I and II loci (especially the *HLA-A*, *-B*, *-C*, *-DRB1*, *-DQB1* and *-DQA1* loci), which are the human Major Histocompatibility Complex (MHC) genes. These genes encode proteins that mediate a critical step of the adaptive immune response, which is the binding of peptides for presentation on the surface of the cellular membrane. The HLA-peptide complex is surveyed by T-cell receptors, which may trigger an immune response when a non-self peptide is identified (Klein and Sato 2000). Balancing selection at HLA loci has been strongly supported by a wide variety of methods, with evidence including an excess of alleles at intermediate frequency with respect to neutral expectations (Hedrick and Thomson 1983), higher non-synonymous to synonymous substitution rate (Hughes and Nei 1988) and *trans*-specific polymorphism (Lawlor *et al.* 1988) (Meyer and Thomson 2001, for a review).

Although balancing selection at HLA genes is well documented, the evidence from most studies is compatible with different mechanisms that are difficult to disentangle: heterozygote advantage (Doherty and Zinkernagel 1975; Takahata and Nei 1990; De Boer *et al.* 2004), frequency-dependent selection (Slade and McCallum 1992; Borghans *et al.* 2004) and selection that varies over time and space (Eizaguirre *et al.* 2012) have all been proposed to act on the HLA genes.

Interestingly, these selective regimes are theoretically compatible with both increased or reduced population differentiation. For example, the coevolution between HLA and pathogens could create a mechanism of frequency-dependent selection, or rare allele advantage. Under this scenario, rare HLA alleles would be advantageous, since few pathogens would have evolved resistance to them (Meyer and Thomson 2001). Rare allele advantage is expected to increase the effective migration rate: migrants will often be rare in the population they arrive to, and thus will be advantageous and increase in frequency in the new population (Schierup *et al.* 2000; Muirhead 2001). Therefore, this regime of balancing selection is expected to reduce population differentiation.

However, there are also cases where balancing selection is associated to an increase in population differentiation. For example, de Filippo *et al.* (2016) identified four non-HLA loci which were under balancing selection in Africans, but positive selection in Eurasians, resulting in large frequency differences between these regions. In the case of HLA genes, selection may be population-specific, with the different sets of pathogens in each population selecting locally advantageous HLA variants. Under these scenarios we expect an increase in population differentiation.

Evidence in support of population-specific pathogen selection for humans comes from the finding that HLA and pathogen diversities across populations are correlated (Prugnolle *et al.* 2005), and from theoretical studies showing that population-specific pathogen selection models of balancing selection provide a better explanation for observed HLA variation than heterozygote advantage (Hedrick 2002; Borghans *et al.* 2004).

Pathogen-driven selection implies that specific HLA alleles are more effective in presenting antigens of certain pathogens, to which the population is exposed. Support for this assumption comes from associations between disease susceptibility, resistance or progression with genetic variation at HLA. For example, variants in *HLA-B* are associated to the progression to clinical disease after HIV infection (The International HIV Controllers Study 2010), variants in *HLA-C*, *-DP* and *-DQ* are associated to clearance of hepatitis B virus (Hu *et al.* 2013), alleles in *HLA-DRB1* are associated to susceptibility or resistance to leprosy (Vanderborght *et al.* 2007) and alleles at *HLA-B*, *-DQ* and *-DR* are associated to resistance to severe malaria (Hill *et al.* 1991).

Previous studies which measured population differentiation at the HLA genes found evidence ranging from increased to reduced population differentiation. For example, Meyer *et al.* (2006) found no significant difference between differentiation at HLA genes and a set of neutral markers used as a control (microsatellites), while Sanchez-Mazas (2007) found lower differentiation at HLA loci than in their genome-wide control (microsatellites and RFLPs). A limitation of these studies is that differences between the neutral genetic markers and the sequence data used for HLA genes introduce confounding variables, making it difficult to determine the roles of selection or characteristics inherent to the marker (*e.g.*, mutation rate and diversity). Another study compared differentiation on markers of the same type (microsatellites) located at HLA genes or near them and those located in other genomic regions, which serve as controls (Nunes 2011). This study found increased differentiation in regions near HLA genes. Nonetheless, some issues remain unresolved: Nunes (2011) was mainly interested in native American populations, and used a limited number of markers. Furthermore, the complexity of the mutational mechanism of microsatellites complicates the interpretation of results.

For non-model organisms a similarly wide array of results have been found, with the MHC region (which contains genes homologous to HLA) showing either equal (Miller *et al.* 2010), higher (Loiseau *et al.* 2009; Oliver *et al.* 2009; Cammen *et al.* 2011) or lower (McCairns *et al.* 2011) differentiation than genome-wide averages. These contrasting results could be due to differences in selective regimes among species, or even to variation in selection among genes within a species (Čížková *et al.* 2011).

In summary, it remains unclear whether balancing selection on HLA genes drives increased differentiation due to selection favoring adaptation to locally occurring pathogens, or whether it results in decreased genetic differentiation due to the maintenance of shared polymorphisms among populations.

Here, we revisit the question of population differentiation at the HLA genes using sequence data from worldwide human populations. We investigate variation at SNPs, which have the advantage of allowing the use of genomic data as an empirical control for HLA SNPs, assuming similar mutation rates for SNPs in the MHC region and the reminder of the genome. Differently from scans that seek genome-wide significance for specific SNPs, we *a priori* define a set of putatively selected SNPs to be surveyed (those within or close to HLA genes). We relate differences in F${}_{\text{ST}}$ between HLA and non-HLA SNPs to the degree of polymorphism in each of these groups, drawing on recent findings concerning the constraints imposed by allele frequencies on measures of differentiation. Finally, we perform simulations and find a plausible selective regime that reproduces our results.

## Materials and Methods

### SNP Data

SNP genotypes were acquired from the integrated Variant Call Format (VCF) files from phase 3 of the 1000 Genomes Project (1000G) (The 1000 Genomes Project Consortium 2015), which are available at ftp://ftp-trace.ncbi.nih.gov/1000genomes/ftp/release/20130502/.

#### Sample filters:

The 1000G Phase 3 sequenced 2504 individuals from 26 populations. Six of those populations are admixed: Americans of African Ancestry in the Southest USA (ASW); individuals of Mexican Ancestry from Los Angeles in the USA (MXL); Puerto Ricans from Puerto Rico (PUR); Colombians from Mendellín, Colombia (CLM); African Caribbeans from Barbados (ACB); and Peruvians from Lima, Peru (PEL). We focus on the effect of balancing selection on differentiation of diverging populations, and therefore we excluded admixed populations from our analyses. After excluding admixed populations, a total of 2000 individuals in 20 populations were kept (see Table S1).

#### Site filters:

The 1000 Genomes phase 3 data includes variants discovered via high coverage exome targeted resequencing as well as low coverage whole genome resequencing. Because of the higher coverage in exonic regions, all comparisons between HLA SNPs and non-HLA SNPs (which we treated as a control set) were made within the same functional category (*e.g.*, intronic or exonic). To this end each SNP was annotated using ANNOVAR (Wang *et al.* 2010). Our findings for differences in F${}_{\text{ST}}$ between HLA and non-HLA SNPs were qualitatively the same when using either exonic or intronic regions, so throughout the paper we focus on the results for exonic regions.

Single nucleotide variants with more than 2 alleles in the 1000G data were excluded from our analyses, to keep consistency with our analytical results on the relationship between minor allele frequency and population differentiation, which are defined for biallelic variants. In total, 954,219 SNPs in 1000G are exonic and biallelic. Of these, 832,892 are polymorphic in the set of populations we analyzed, with 525 located within HLA genes, and 1193 located within peri-HLA genes (see “Definition of HLA, peri-HLA and control regions” section, below).

The use of a single reference genome to map Next Generation Sequencing (NGS) reads creates mapping bias at some HLA SNPs in the 1000 Genomes Project phase 1 dataset (Brandt *et al.* 2015). Another potential source of error in NGS data for the HLA region is due to copy number variation of some genes, which could cause alleles to be under or over-called, if there are more or less copies of the gene present in the reference genome, respectively. We therefore excluded the SNPs within the HLA genes which have unreliable frequency estimates in the 1000 Genomes phase 1 dataset, compared to gold-standard sequencing data generated by Sanger sequencing (Brandt *et al.* 2015). After applying this filter, 38 out of 525 exonic biallelic SNPs in the HLA genes were excluded (Table S3). In total, 487 SNPs were kept in the HLA group, 1193 in the peri-HLA group, and 831,174 in the control group.

In the present study we analyze this filtered version of the 1000 Genomes phase 3 data instead of phase 1 or Sanger sequencing data analyzed in Brandt *et al.* (2015) because it has a larger sample size and includes more populations. Also, SNPs identified as unreliable in the 1000 Genomes phase 1 data were generally the same across different populations, which supports the application of this filter to phase 3 data used here.

### Estimation of F${}_{\text{ST}}$ and minor allele frequency

Population differentiation was calculated as the proportion of variance in allele frequencies among populations (*a*), relative to the total genetic variance (a+b+c, with *b* and *c* referring to the variance components between individuals within populations and between gametes within individuals, respectively):

$$F_{ST}=\frac{a}{a+b+c}$$ (1)

To obtain F${}_{\text{ST}}$ values we used the Weir and Cockerham (1984) estimator implemented in VCFtools v0.1.14 (Danecek *et al.* 2011). The Weir and Cockerham (1984) estimator was chosen because it is unbiased when sample sizes are large and similar, as in the case of our dataset. F${}_{\text{ST}}$ was calculated per SNP i) over all populations and ii) for pairs of populations. In the first case, all sites that were variable in at least one of the 20 populations were used to estimate F${}_{\text{ST}}.$ In the second case, only sites that were variable in at least one population in the pair were used to estimate F${}_{\text{ST}}$ between the two populations.

When summarizing F${}_{\text{ST}}$ over multiple SNPs, we compared two approaches: i) computing a simple average of F${}_{\text{ST}}$ at individual SNPs and ii) using the “ratio of averages” approach, suggested by Reynolds *et al.* (1983), in which we first estimate the numerator (*a*) and denominator (a+b+c) of F${}_{\text{ST}}$ for each SNP, and then compute the averages of *a* and a+b+c for the desired set of SNPs, and finally compute the ratio of both averages. This second approach provides the least biased estimate of F${}_{\text{ST}},$ whereas performing a simple average of the F${}_{\text{ST}}$s of each SNP can lead to an underestimation of differentiation, especially in datasets rich in rare variants (Reynolds *et al.* 1983; Bhatia *et al.* 2013). Unless otherwise stated, we used the “ratio of averages” approach to compute F${}_{\text{ST}}.$ Variance components (*a* and a+b+c) were obtained using a minor modification of the VCFtools source code described in the Extended Materials and Methods.

Allele frequency (AF) was calculated per SNP, per population using VCFtools v0.1.14b (Danecek *et al.* 2011). Minor allele frequency (MAF) was calculated as 1−AF, when AF>0.5. Global minor allele frequency (over all populations) was calculated as the frequency of the rarest allele in the pooled sample of 2000 individuals.

### Definition of HLA, peri-HLA and control regions

We define “HLA SNPs” as those contained within the coding sequence of the classical HLA genes *HLA-A*, *-B*, *-C*, *-DRA*, *-DRB1*, *-DQA*, *-DQB1*. Previous studies of *HLA-DPA1* and *-DPB1* found weak or no evidence of balancing selection (Solberg *et al.* 2008; Begovich *et al.* 2001), and even instances of directional selection (Hollenbach *et al.* 2001), making them inappropriate for our question concerning the role of balancing selection on differentiation at HLA loci. Accordingly, our analyses also showed that population differentiation at *HLA-DPA1* and *HLA-DPB1* genes is different from that of other HLA genes (see Results and Figure S4). Therefore, those loci were excluded from our analysis unless otherwise mentioned.

Peri-HLA genes were defined as those that flank the HLA genes and have higher diversity relative to the average of chromosome 6 (Mendes 2013), indicating that their increased polymorphism is driven by hitchhiking to the strongly selected HLA loci (Table S2). These genes are located 119kb to 256kb from the closest HLA locus. All SNPs outside both the HLA and peri-HLA genes comprised the control group.

### Statistical test of F${}_{\mathrm{ST}}$ differences

When comparing F${}_{\text{ST}}$ values between HLA and control SNPs, we control for the fact that HLA SNPs are located within a small set of genes, resulting in higher linkage disequilibrium (LD) and statistical non-independence than for SNPs in the control group, which comprise a genomewide set. To account for this effect, we designed a strategy to sample the control SNPs in a way that approximates the LD structure among the HLA SNPs.

To do this, we first randomly sampled an exonic SNP and extracted the MAF of all the other exonic SNPs within that gene. Each sampled SNP was assigned to one of six MAF bins: one bin for rare SNPs (0-0.01), and five bins with approximate width of 0.1 (0.01-0.1, 0.1-0.2, 0.2-0.3, 0.3-0.4, 0.4-0.5). This combination of bins allowed us to sample SNPs from various frequency classes, without creating excessively sparse intervals. We then went on to draw another random exonic SNP (from another gene), repeating this process until for each MAF bin we had the same number of SNPs as for the HLA SNPs. If a specific MAF bin was complete (*i.e.*, contained the number of HLA SNPs in that MAF interval) before others, we only kept SNPs from the sampled gene for the MAF bins which were still incomplete (see Figure S5). As a result of this sampling scheme, the control sample contained SNPs collected from a smaller subset of genes than if SNPs were randomly drawn from the genome. In this way our control SNPs had similar properties to the HLA SNPs: high LD among the sampled SNPs and matching MAF distribution. This entire process was repeated 1000 times, providing 1000 sets of control SNPs.

We compared the F${}_{\text{ST}}$ distribution of each of the 1000 sets of control SNPs to that of the HLA SNPs, applying a Mann-Whitney test. We recorded the number of comparisons where the difference between F${}_{\text{ST}}$ distributions was significant (p < 0.05).

### Haplotype level analyses

In addition to SNP based analyses, we also investigated population differentiation when alleles are defined by the coding sequence of each HLA gene (classically referred to as an “HLA allele”). We treat these analyses as “haplotype level”, where haplotypes are defined by a combination of SNPs along an HLA gene (*i.e.*, each allele in these analyses is an intragenic haplotype).

Phasing of SNPs in extremely variable regions like the HLA is complicated by the high SNP density and polymorphism. Therefore, rather than estimating intragenic haplotypes directly from the SNP data, we used a publicly available dataset which provides HLA allele calls for samples in the 1000G data based on Sanger sequencing (Gourraud *et al.* 2014), available at the dbMHC website (ftp://ftp.1000genomes.ebi.ac.uk/vol1/ftp/technical/working/20140725_hla_genotypes/20140702_hla_diversity.txt).

We restricted these haplotype level analyses to *HLA-A*, *-B*, *-C*, *HLA-DRB1* and *-DQB1*, which are reported in Gourraud *et al.* (2014). HLA allele calls were coded so as to only distinguish alleles with nonsynonymous differences (*i.e.*, only the first two fields of the allele names were used, as described in the HLA nomenclature system) (Marsh *et al.* (2010)). When the HLA typing method generated an ambiguous allele call, the most common allele was chosen.

F${}_{\text{ST}}$ values of multiallelic HLA haplotypes can’t be directly compared to those of biallelic SNPs because multiallelic loci tend to have lower allele frequencies, which constrains the maximum value of F${}_{\text{ST}}$ (Jakobsson *et al.* 2013). To allow F${}_{\text{ST}}$ values at the HLA haplotypes to be compared to this null distribution, we recoded each HLA gene as a series of biallelic loci. This recoding was done by treating each allele at each gene as “allele 1”, and all other alleles as “allele 2”. The Weir and Cockerham (1984) F${}_{\text{ST}}$ estimator was then computed as described for SNPs, using the *wc* function of the hierfstat R package (Goudet 2005).

### Data availability

Supplementary material is available on Figshare. Extended Materials and Methods shows the modification of VCFtools source code to get variance components of F${}_{\text{ST}}$. File S1 shows the constraints of minor allele frequency on F${}_{\text{ST}}.$ File S2 is a detailed description of the balancing selection simulations. Figure S4 shows F${}_{\text{ST}}$ as a function of MAF for each HLA gene. Figure S5 is a flowchart describing the steps of the resampling algorithm used to account for LD among HLA SNPs. Figure S6 shows distributions of F${}_{\text{ST}}$ in bins of MAF lower than 0.01. Figure S7 shows distributions of F${}_{\text{ST}}$ per MAF bin, including outliers. Figure S8 shows hierarchical population differentiation (F${}_{\text{CT}}$ and F${}_{\text{SC}}$) along chromosome 6. Table S1 shows sample sizes per population. Table S2 is a list of peri-HLA genes and their genomic coordinates. Table S3 is a list of sites excluded due to excess of genotyping errors. Table S4 shows the results of the statistical test of differences in F${}_{\text{ST}}$ distributions in HLA and non-HLA SNPs, accounting for LD. Supplemental material available at Figshare: https://doi.org/10.25387/g3.6671753.

## Results

### Higher F${}_{\mathrm{ST}}$ in HLA genes

**Figure 1:**
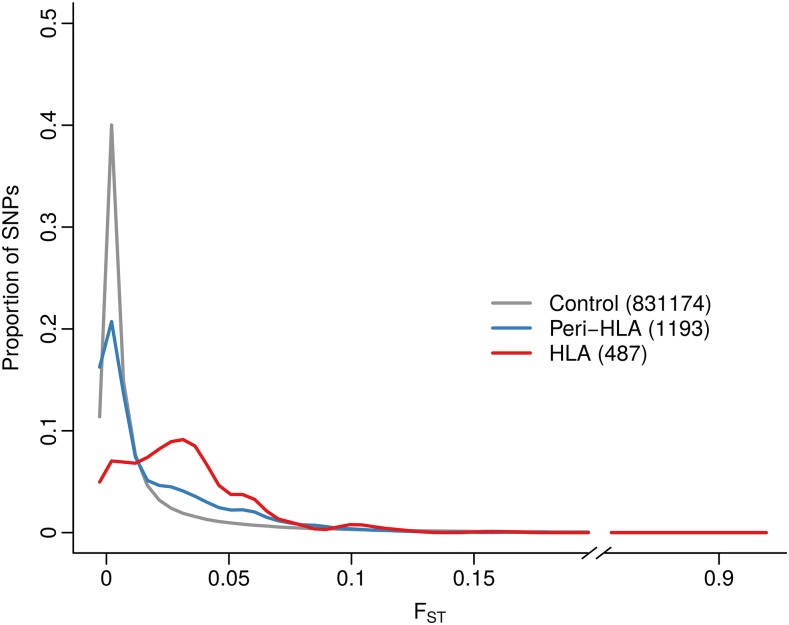
Distribution of F${}_{\text{ST}}$ values for exonic SNPs from the following groups: outside HLA and peri-HLA regions (control group), within HLA genes and within peri-HLA genes. The number of SNPs in each group is shown in parentheses. Density was estimated for each category of SNPs at 190 points equally spaced along the range of observed F${}_{\text{ST}}$ values, using a Gaussian kernel. Density at each point was normalized by dividing by the sum of densities at each category, such that the height represents the proportion of SNPs with that F${}_{\text{ST}}$ value.

Initially we compared the distribution of F${}_{\text{ST}}$ among SNPs from the HLA, peri-HLA and control groups (Figure 1).

The distribution of F${}_{\text{ST}}$ of SNPs within HLA genes is shifted toward higher values (median = 0.027) compared to control SNPs (median=$9.2\times 10^{-5}$), and the distributions are significantly different (p-value $<10^{-16},$ two-tailed Mann-Whitney test).

Theory predicts that balancing selection affects only a narrow genomic region, the size of which is defined by the intensity of selection and the recombination rate (Charlesworth *et al.* 1997). To evaluate if the increased differentiation at coding SNPs within HLA genes was also observed in loci that flank the HLA, we applied the same test to the peri-HLA genes. Although the shift is less striking than for HLA SNPs, peri-HLA SNPs also have an F${}_{\text{ST}}$ distribution with significantly higher values (median = 0.006, p-value $<10^{-16},$ two-tailed Mann-Whitney test; Figure 1).

### Lower F${}_{\mathrm{ST}}$ in HLA when accounting for MAF

**Figure 2:**
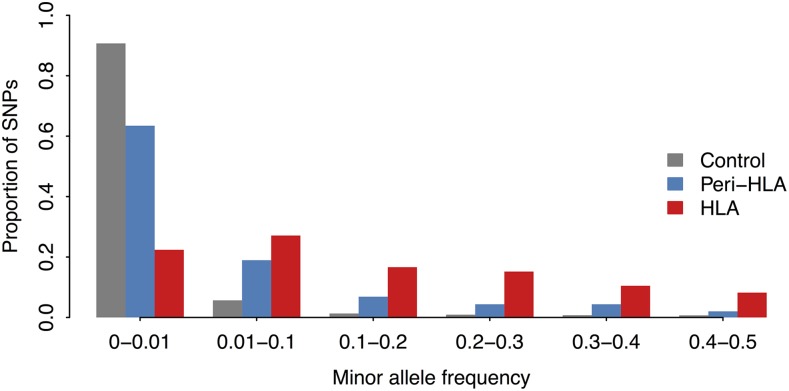
Distribution of minor allele frequency (MAF) for exonic control, peri-HLA genes and HLA genes. MAF of SNPs at the HLA and peri-HLA genes is higher compared to other genes.

The effect of balancing selection is to shift the site frequency spectrum (SFS) of selected loci toward an excess of intermediate frequency variants. This shift is precisely what we see in the data, with the SFS for HLA SNPs showing a shift to intermediate frequencies compared to control SNPs. The peri-HLA SNPs occupy an intermediate position in the SFS (Figure 2).

**Figure 3:**
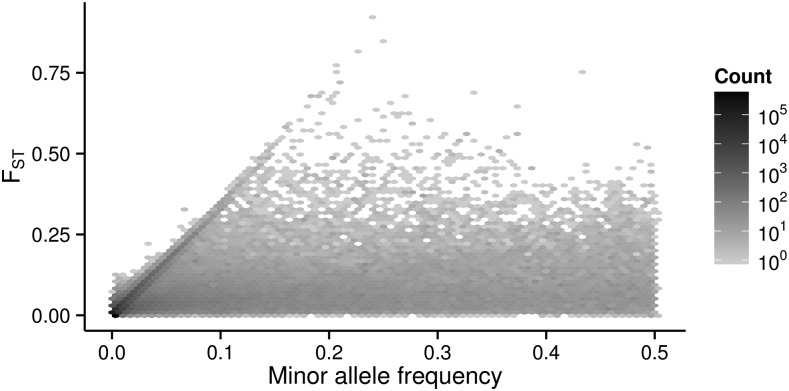
Population differentiation, measured by F${}_{\text{ST}},$ as a function of minor allele frequency at biallelic exonic SNPs from the 1000 Genomes Project phase 3 data.

Constraints imposed by allele frequencies on F${}_{\text{ST}}$ have been a topic of recent investigation (Roesti *et al.* 2012; Maruki *et al.* 2012; Elhaik 2012; Jakobsson *et al.* 2013; Edge and Rosenberg 2014; Alcala and Rosenberg 2017), and it has been shown that SNPs with very low minor allele frequencies (MAF) are bounded to low F${}_{\text{ST}}$ values. This relationship between MAF and F${}_{\text{ST}}$ is empirically illustrated for the 1000 Genomes exome data in Figure 3, which shows that F${}_{\text{ST}}$ is constrained to low values mainly in the range of low MAF (up to ∼0.15 in the 1000G dataset). For MAF values above 0.15 the constraint is no longer evident. In File S1, we analytically show the relationship between MAF and the maximum possible value of F${}_{\text{ST}},$ which has also been shown by Alcala and Rosenberg (2017).

This relationship between MAF and maximum F${}_{\text{ST}}$ suggests that the large number of rare variants in the 1000G dataset, and the relative paucity of low MAF variants in the HLA SNPs, can explain the differences in F${}_{\text{ST}}$ distributions between control and HLA SNPs seen in Figure 1.

**Figure 4:**
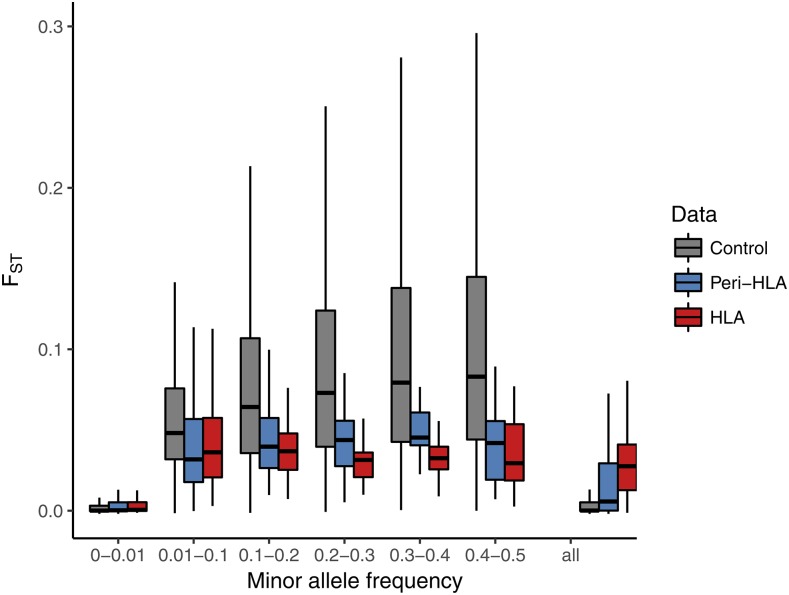
F${}_{\text{ST}}$ distributions per minor allele frequency (MAF) bin. HLA and peri-HLA SNPs show lower F${}_{\text{ST}}$ than control SNPs in all bins with MAF >0.01. Outliers (points above the 3^rd^ quartile by 1.5 times the interquartile range, or below the 1^st^ quartile by the same amount) were removed from figure, but not from statistical test, for better visualization. Figure S7 shows F${}_{\text{ST}}$ distributions including outliers.

To account for the effects of differences in SFS between HLA and control SNPs when contrasting population differentiation among those groups, we compared the F${}_{\text{ST}}$ of HLA and control SNPs within bins of MAF values (Figure 4). Contrary to what we observed without controlling for MAF, we now find that HLA and peri-HLA SNPs have significantly lower F${}_{\text{ST}}$ than at the control SNPs (Mann-Whitney two-tailed test p-value $<10^{-5}$ for all bins of MAF > 0.01). The bin with MAF < 0.01 shows a similar pattern when further split into smaller bins of MAF (Figure S6).

This approach used SNPs outside the HLA and peri-HLA genes as controls for the HLA SNPs. However, because the HLA SNPs are located in 5 genes, they are not independent, due to both intra and inter-locus associations. As a consequence, our p-values could be inflated by treating a set of correlated SNPs as independent, and comparing them to a set of control SNPs which are in their majority independent. We controlled for this non-independence using a resampling approach, in which sets of linked SNPs were sampled from our control group, and their F${}_{\text{ST}}$ values were compared to those from the HLA SNPs, at each MAF bin (see Materials and Methods). After controlling for the non-independence of HLA SNPs, we confirmed that F${}_{\text{ST}}$ at HLA SNPs was significantly lower than at the resampled SNPs, at all bins of MAF higher than 0.01 (Table S4).

If instead of comparing F${}_{\text{ST}}$ distributions, one is interested in comparing average F${}_{\text{ST}}$ between different genomic regions, it is again necessary to account for the constraint of MAF on F${}_{\text{ST}}$. Computing a simple average F${}_{\text{ST}}$ of individual SNPs creates a bias leading to an underestimation of F${}_{\text{ST}},$ the effect of the bias being more pronounced when there are more rare variants in the dataset (Bhatia *et al.* 2013). To control for the effect of rare variants reducing average F${}_{\text{ST}},$ we estimate average F${}_{\text{ST}}$ for multiple loci by separately computing averages of the numerator and denominator of the AMOVA-based F${}_{\text{ST}}$ estimator, and then taking their ratio (see Methods). Reynolds *et al.* (1983) and Weir and Cockerham (1984) showed that this is the least biased estimate of average F${}_{\text{ST}}$ across multiple loci. This “ratio of averages” approach downweights the contribution of variants with low MAF to the average F${}_{\text{ST}},$ resulting in higher average F${}_{\text{ST}}.$

We explored how these different averaging methods impact the F${}_{\text{ST}}$ at HLA genes, and found that the “ratio of averages” approach (which controls for MAF) results in substantially lower average F${}_{\text{ST}}$ at the HLA SNPs (0.04) than in the control SNPs (0.09). In stark contrast, using the average of individual loci F${}_{\text{ST}}$ (*i.e.*, an “average of ratios”), we found higher F${}_{\text{ST}}$ values for the HLA SNPs (0.03) than genome-wide (0.01), as in our initial analysis that did not account for MAF (Figure 1). The effect of different F${}_{\text{ST}}$ averaging approaches further emphasizes the importance of accounting for the differences between the site frequency spectrum of HLA and control SNPs when assessing population differentiation.

#### HLA-DP genes:

The classical *HLA-DPA1* and *-DPB1* genes were excluded from the previous analysis because they show weak or no evidence of balancing selection (Solberg *et al.* 2008; Begovich *et al.* 2001), and some evidence of directional selection (supported by the observation that within individual populations a small number of alleles are present at a high frequency) (Hollenbach *et al.* 2001).

Consistently with being under a different selective regime, *HLA-DPA1* and *-DPB1* show a pattern of population differentiation which is different from the other classical HLA loci: F${}_{\text{ST}}$ at these genes is higher than in the control SNPs, even when minor allele frequency is controlled for (Figure S4).

#### Contrasting F${}_{\mathrm{ST}}$ of HLA SNPs and haplotypes:

Next, we explored population differentiation at the haplotype level (*i.e.*, with alleles defined by the coding sequence of each HLA gene). Haplotype level analyses were motivated by the idea that the fitness of individuals is more likely to be determined by the combination of SNPs they carry in a gene, rather than by individual SNPs, since it is the combination of SNPs that determines the peptides which HLA molecules present.

To compare F${}_{\text{ST}}$ for HLA haplotypes to a null distribution, we recoded each HLA gene as a biallelic locus (see Materials and Methods). Population differentiation at the recoded HLA haplotypes was then compared to differentiation at control SNPs and HLA SNPs, while controlling for minor allele frequency, as was done for SNPs.

**Figure 5:**
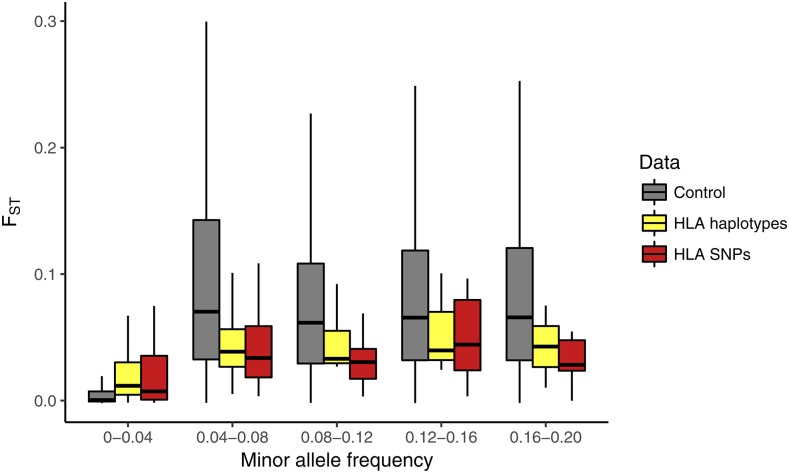
Population differentiation (F${}_{\text{ST}}$) as a function of minor allele frequency for the exonic control SNPs (gray), for exonic HLA SNPs (red) and for the haplotype-level HLA alleles (yellow) after recoding as biallelic (see Materials and Methods). No haplotypes had frequency higher than 0.2, therefore MAF bins were redefined.

HLA haplotypes show population differentiation intermediate between HLA SNPs and control SNPs. Differentiation at HLA haplotypes was not significantly different from HLA SNPs in any MAF bin, and was not significantly different from control SNPs, except for the two MAF bins between 0 and 0.08 (Figure 5). Thus, despite the existence of haplotypes which are specific to certain world regions, when the average MAF is considered and global F${}_{\text{ST}}$ is quantified, the degree of differentiation of HLA haplotypes tends to be lower than that of control SNPs.

It is worth noting that ambiguous HLA haplotype calls (*i.e.*, instances where the typing method provided a set of possible allele calls) were resolved by choosing the assignment that minimized population differentiation. Thus, a more reliable assessment of haplotype-level differentiation will require less ambiguous haplotype calls.

### F${}_{\mathrm{ST}}$ at HLA SNPs depends on divergence times

Our previous analyses examined global F${}_{\text{ST}},$ which captures patterns of differentiation among all 20 populations retained from the 1000 genomes full dataset. Next, we asked how specific populations contributed to our findings. In order to investigate this question, and to evaluate how the geographical scale (within and among continent comparisons) influences differentiation at the HLA, we analyzed F${}_{\text{ST}}$ between all pairs of populations.

**Figure 6:**
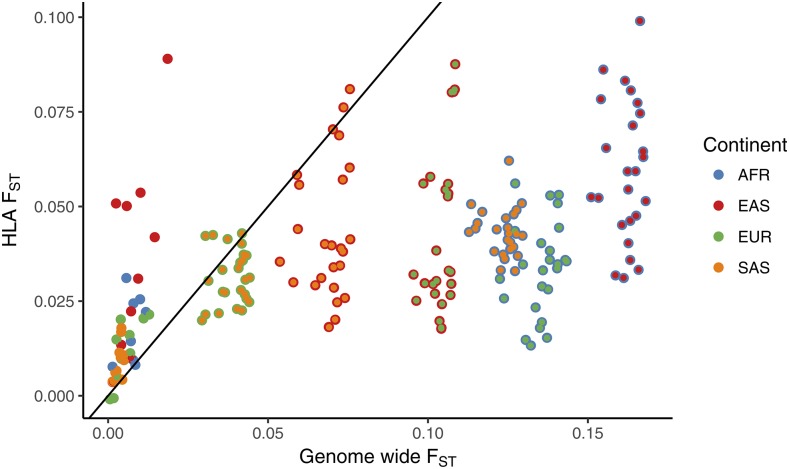
Average F${}_{\text{ST}}$ at the HLA and at the control SNPs for each pair of populations. Points with a single color represent pairs of populations from the same continent. Average F${}_{\text{ST}}$ was computed using the ”ratio of averages” approach, described in the main text.

We found that the lower differentiation at HLA SNPs as compared to control SNPs seen in our previous results (Figure 4) in seen for highly diverged populations (Figure 6) (*i.e.*, contrasts involving populations from different continents).

However, population pairs within the same continent show higher differentiation at the HLA SNPs compared to control SNPs. We also observed this effect in an independent analysis of 51 populations from the Human Genome Diversity Project (HGDP) SNP dataset (Li *et al.* 2008), with F${}_{\text{SC}}$ (differentiation among populations within continents) values showing a peak in the MHC region (Figure S8).

## Discussion

### Population differentiation at HLA SNPs

In an overall analysis of F${}_{\text{ST}}$ among worldwide populations, we found significantly decreased genetic differentiation at HLA SNPs. We have shown that this result is critically dependent on the use of methods which appropriately account for the properties of the site frequency spectrum of HLA genes.

The decreased differentiation at HLA genes counters the expectation of a model of adaptation to local pathogens driving differentiation. However, we found that the overall pattern of lower differentiation reveals a greater complexity when we compare populations with different divergence times.

### Mind the MAF

Our results highlight the importance of accounting for minor allele frequency (MAF) when comparing F${}_{\text{ST}}$ values for different sets of SNPs, as previously shown by others (Roesti *et al.* 2012; Maruki *et al.* 2012; Elhaik 2012; Jakobsson *et al.* 2013; Edge and Rosenberg 2014; Wang 2015; Alcala and Rosenberg 2017). Not accounting for MAF leads to an underestimation of population differentiation for sets of SNPs rich in rare variants. When comparing sets of SNPs from genomic regions with different MAF distributions, the constraint of MAF on F${}_{\text{ST}}$ may result in a misleading interpretation of the selective regime acting on each region.

We empirically demonstrated the constraint of F${}_{\text{ST}}$ by MAF in the 1000 Genomes (Figure 3), and Alcala and Rosenberg (2017, Figure 7) have demonstrated the same effect in the HGDP dataset (Li *et al.* 2008). In Alcala and Rosenberg (2017), the constraint is given in terms of the frequency of the most frequent allele, and it is strongest when the frequency of the most frequent allele is above 0.85, which is equivalent to MAF below 0.15.

**Figure 7:**
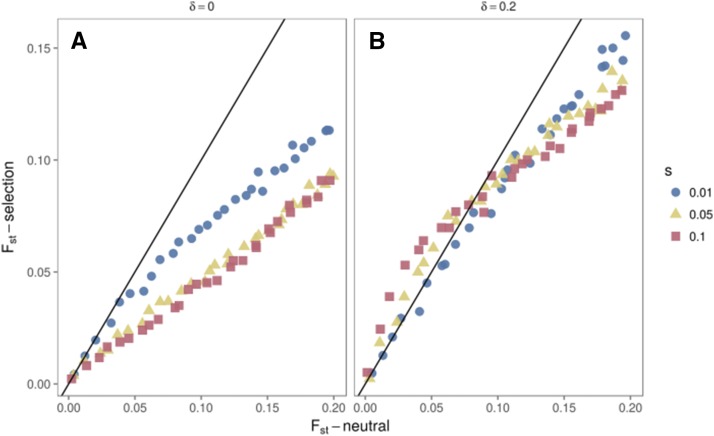
Relation between $F_{ST}$ under neutrality and balancing selection. The black line indicates F${}_{\text{ST}}$neutral = F${}_{\text{ST}}$selection. (A) daughter populations remain under the same regime of overdominance as the ancestral population (shared overdominance). (B) One of the daughter populations experiences a shift in the fitness values (divergent overdominance), remaining under overdominance but with a new equilibrium value (changed by a value of δ=0.2). Under divergent overdominance, for recent divergence times we find balancing selection can transiently increase population differentiation, so long as selection is strong (s = 0.05 or greater, $N_{e}=1000$).

Our results differ slightly from Alcala and Rosenberg (2017) in the slope of the diagonal that shows the constraint of F${}_{\text{ST}}$ at low MAF, which is steeper in Alcala and Rosenberg (2017), Figure 7. Also, our results presented in Figure 3 show more SNPs along that diagonal, and some seemingly above where the theoretical maximum would be, especially at low MAF. Although we have not directly tested the causes of these differences, we point out two potential explanations. First, different F${}_{\text{ST}}$ estimators were used: we estimated F${}_{\text{ST}}$ according to Weir and Cockerham (1984), while Alcala and Rosenberg (2017) calculated parametric F${}_{\text{ST}}.$ Second, we have computed F${}_{\text{ST}}$ among 20 populations in four major geographic groups (listed in Table S1), while Alcala and Rosenberg (2017) compared seven geographic regions, therefore excluding within-region comparisons with the lowest F${}_{\text{ST}}$ values.

Alcala and Rosenberg (2017) also arrived at the same results for the upper boundary of F${}_{\text{ST}}$ as a function of allele frequency in biallelic markers as we present on Supplemental File S1. Their work presents a more general investigation of the constraints of the frequency of the most frequent allele on F${}_{\text{ST}},$ using simulations under different migration models. Alcala and Rosenberg (2017) discuss the effect of this constraint in reducing the power of outlier tests which use high F${}_{\text{ST}}$ as a signature of local positive selection. Here we emphasize the effect of this constraint on interpreting F${}_{\text{ST}}$ values at regions under balancing selection, where the depletion of rare variants leads to higher overall population differentiation than in other genomic regions, if no control for MAF is performed. However, when variants with similar MAF are compared, population differentiation in the region under balancing selection is actually lower, as we show for SNPs within HLA genes.

While the constraint of MAF on F${}_{\text{ST}}$ strongly affects differentiation at regions under positive and balancing selection, it is less problematic in regions under purifying selection. Under this selective regime, both an enrichment of low frequency variants and low population differentiation are expected. Since population differentiation in low frequency variants is constrained to low values, the relationship between MAF and F${}_{\text{ST}}$ leads both signatures in the same direction. This effect has been demonstrated in *Drosophila melanogaster* (Jackson *et al.* 2014).

### Contrast to previous studies

Two classical HLA genes, *HLA-DPA1* and *HLA-DPB1*, were exceptions to our finding of lower population differentiation at HLA SNPs. The SNPs in these genes showed higher population differentiation, even when MAF was accounted for (Figure S4). This finding supports previous evidence that HLA-DP genes are under a different selective regime from other classical HLA genes. For example, a genomic scan performed by Bhatia *et al.* (2011) also found a SNP near a HLA-DP gene with unusually high population differentiation (rs2179915, which is 30kb from *HLA-DPA2*). Similarly, Barreiro *et al.* (2008) found unusually high F${}_{\text{ST}}$ at the *HLA-DPB2* locus, applying a threshold value of $F_{\text{ST}}>0.65,$ which is in the tail of F${}_{\text{ST}}$ distributions at any MAF bin. Together, these results indicate that the HLA-DP genes are not under balancing selection, but rather under directional selection that differs among populations. One possibility is that HLA-DP has suffered a recent change in selective regimes, in which variation maintained by balancing selection becomes positively selected in specific populations, as has been shown in other loci (de Filippo *et al.* 2016).

The studies of Bhatia *et al.* (2013) and Barreiro *et al.* (2008) were designed with an emphasis on the detection of extremely high differentiation, and thus did not capture the pattern of low differentiation which is characteristic of most HLA loci. Hofer *et al.* (2012), on the other hand, used an approach where regions of adjacent SNPs with extreme F${}_{\text{ST}}$ were scanned for, and found unusually low F${}_{\text{ST}}$ for *HLA-C*. The method used by Hofer *et al.* (2012) evaluates F${}_{\text{ST}}$ as a function of the heterozygosity between populations, which for biallelic markers is equivalent to the correction for MAF we applied here. These results show that when a test is designed to account for the possibility of unusually low F${}_{\text{ST}}$ values, and when the effect of minor allele frequency (or heterozygosity) on F${}_{\text{ST}}$ is accounted for, a signature of low differentiation which would otherwise not be detected can be found.

### Divergence time effect

By taking advantage of multiple populations made available by the 1000 Genomes project, we also examined if the excess of low differentiation at HLA SNPs holds at all timescales of differentiation. Interestingly, we find that for population pairs from the same continent F${}_{\text{ST}}$ at HLA SNPs is equal to or higher than in the control SNPs (Figure 6). For highly diverged population pairs (those from different continents), we consistently find lower differentiation among HLA SNPs. This result shows that a specific set of SNPs may differ in how they deviate from the genomic background depending on the timescale of population divergence.

To understand the process driving the increased differentiation for recently diverged populations (Figure 6) we used a simulation approach. Although many different forms of balancing selection could be operating (*e.g.*, frequency-dependent, selection varying over time or space, etc.), here we explore a single and well understood regime, heterozygote advantage (or overdominance).

**Figure 8:**
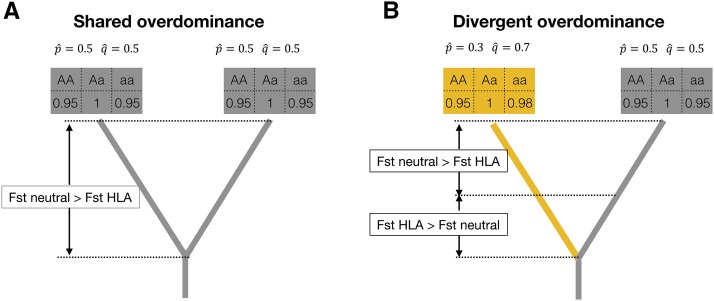
Schematic representation of HLA differentiation under two regimes of overdominance. Each tree represents a population pair experiencing selection according to fitness values presented in the boxes above the tips. The equilibrium frequencies expected under these fitness values are also shown ($\hat{p}$ and $\hat{q}$). In ”shared overdominance” both daughter populations share the same fitness values. In ”divergent overdominance” the fitness values for one of the homozygotes changes in one of the daughter populations, even though the regime is still one of overdominance. In the divergent overdominance scenario, the equilibrium frequencies at the selected site differs among populations, and drives increased differentiation when divergence is recent (F${}_{\text{ST}}$ HLA > F${}_{\text{ST}}$ neutral). For the shared overdominance scenario F${}_{\text{ST}}$ neutral > F${}_{\text{ST}}$ HLA throughout the entire history of population divergence.

First, we simulated a scenario where an ancestral population is under symmetric overdominant selection (equal fitness to both homozygotes), and splits into two daughter populations, both under the same selective regime as the ancestor (details in Supplemental File S2). We refer to this scenario as “shared overdominance”, and find that it results in differentiation between the daughter populations being reduced with respect to neutral expectations (Figure 7A and Figure 8A), in accordance with previous results (Schierup *et al.* 2000).

However, if the two populations remain under overdominant selection but differ for at least one of the homozygote fitness values (a scenario we refer to as “divergent overdominance”), differentiation can be increased with respect to neutrality for small divergence times (Figure 7B and Figure 8B). This result can be understood if we consider that the populations are extremely similar at the time of the split, so the effect of selection will be to favor changes in allele frequency between them (since homozygote fitness and therefore equilibrium frequencies, $f_{eq},$ of selected alleles will differ). As the two populations further diverge, the neutral sites will continue to diverge and will surpass the differentiation for the case of overdominance. Thus, by assuming that the fitness values of an overdominant model can change over time –which is highly plausible if we consider heterogeneity in pathogen populations affecting HLA fitness– increased F${}_{\text{ST}}$ at HLA for recently diverged populations can be explained.

**Figure 9:**
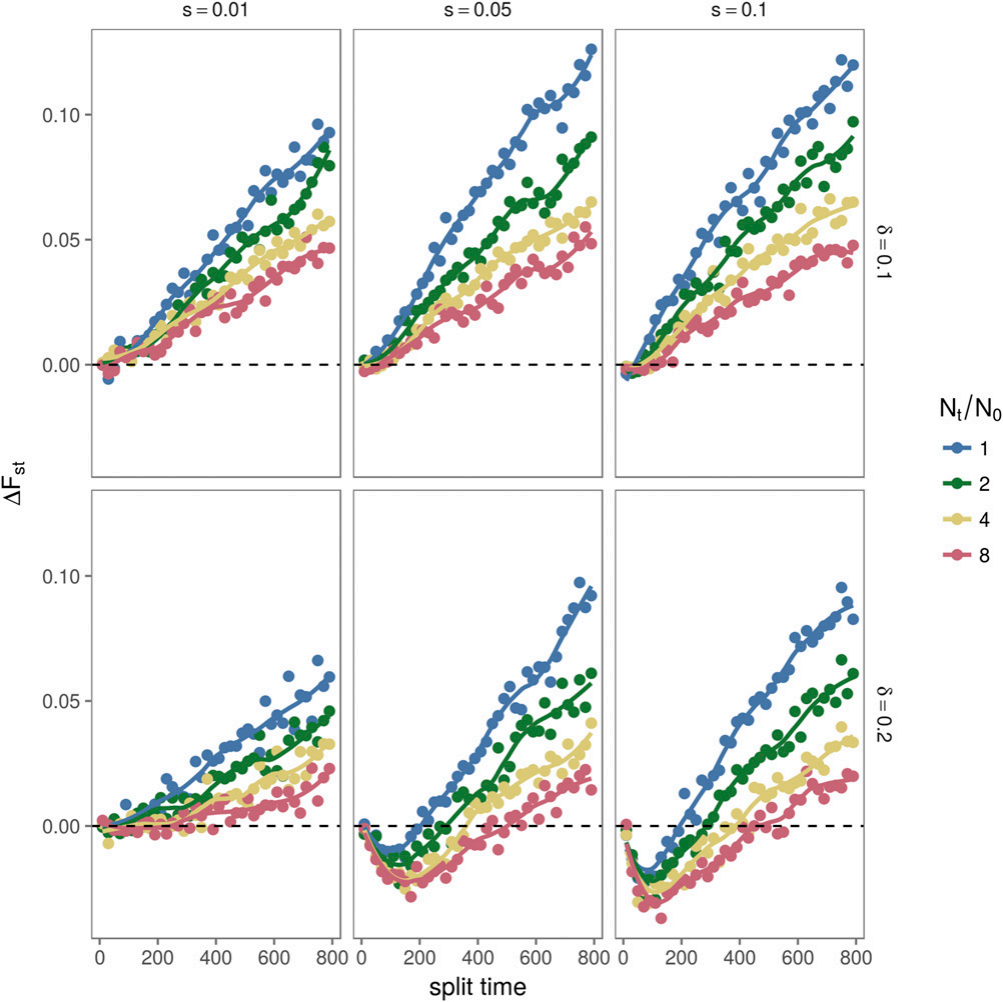
Difference between $F_{ST}$ under neutrality and $F_{ST}$ under overdominance ($\Delta F_{ST}$), for different selective scenarios. For all scenarios shown here, we used an initial equilibrium frequency of 0.5 ($f_{eq}=0.5$), and varied population split times, size fold change ($N_{t}/N_{0}$), selective coefficient (*s*) and difference of equilibrium frequencies after split (*δ*). Notice that for the case of a small change in equilibrium frequency after the split (δ=0.1), the $F_{ST}$ under neutrality is always greater then the $F_{ST}$ under divergent overdominance ($\Delta F_{ST}>0$ for every time after the divergence). For higher values of frequency difference (δ=0.2) and selection coefficient (s>0.05) the $F_{ST}$ under neutrality is smaller than the $F_{ST}$ under divergent overdominance ($\Delta F_{ST}<0$) for times closer to the split of the populations while for times far from the split the $F_{ST}$ under neutrality becomes greater then the $F_{ST}$ under divergent overdominance ($\Delta F_{ST}>0$).

In Figure 9, we further explore parameters of population growth ($N_{t}/N_{0}$) and difference in equilibrium frequencies of alleles under selection after the population split (*δ*). We show that only when the equilibrium frequency difference after the split (*δ*) and the selection coefficient (*s*) are large we recover higher F${}_{\text{ST}}$ under balancing selection than under neutrality (negative $\Delta F_{ST}$) for recent divergence times, and lower F${}_{\text{ST}}$ under balancing selection than under neutrality (positive $\Delta F_{ST}$) for longer divergence times. Different equilibrium frequencies prior to the population split ($f_{eq}$) are explored in Figure S3, and we show that high F${}_{\text{ST}}$ at low divergence times is attained irrespective of $f_{eq}.$

Consistent with our results, recent studies based on signatures of selection other than F${}_{\text{ST}}$ have identified very recent adaptive change at HLA loci (Field *et al.* 2016; Zhou *et al.* 2016), a process which could contribute to differentiation at a local scale without erasing signatures of long-term balancing selection.

### Identifying selective regimes on HLA loci

Selection on HLA genes is now widely understood as a complex process, for which selective regimes may shift over time in intensity, biological targets, or mode of selection. For example, while only long-term balancing selection can account for features of HLA variation such as *trans*-species polymorphism and SFS with a marked excess of intermediate frequency variants (Bitarello *et al.* 2018; Leffler *et al.* 2013), there are also clear signals of recent positive selection, such as local ancestry deviation in recently admixed populations (Meyer *et al.* 2018, Table 3) and a high Singleton Density Score (Field *et al.* 2016). Further, there are recent studies indicating that different selective regimes can act simultaneously on HLA genes, depending on the biological level of organization examined (Lighten *et al.* 2017).

In this study we explored population differentiation at the HLA genes, and show that a divergent overdominance selective regime can reproduce the relationship between population differentiation and divergence time we observed at HLA genes (Figure 7). In Table 1, we summarize our predictions of how other modes of selection could affect population differentiation, and we detail those predictions below.

**Table 1:** Modes of balancing selection and their effects on population differentiation. Balancing selection regimes simulated in this study are shared and divergent overdominance. The last column shows the effects of those regimes on population differentiation measured by F${}_{\text{ST}}$, as well as our predictions of the effects other balancing selection regimes (local and frequency-dependent selection) on F${}_{\text{ST}}$. $F_{ST}sel$ refers to F${}_{\text{ST}}$ under balancing selection and $F_{ST}neu$ refers to F${}_{\text{ST}}$ under neutrality. *A* and *a* represent two different alleles at a biallelic locus. *M* and *m* represent the major and minor allele at a biallelic locus, at any given time, respectively. *p* represents the frequency of the most frequent allele (*M*). *s* and *t* are selection coefficients, and *h* is the dominance coefficient. Subscripts 1 and 2 represent population specific parameters

Mode of balancing selection (reference)	Population 1 fitness			Population 2 fitness			Difference in F${}_{\text{ST}}$ between selected and neutral SNPs
	AA	Aa	aa	AA	Aa	aa	
Shared overdominance (Figure 7A)	1−s	1	1−t	1−s	1	1−t	$F_{ST}sel<F_{ST}neu$
Divergent overdominance (Figure 7B)	$1-s_{1}$	1	$1-t_{1}$	$1-s_{2}$	1	$1-t_{2}$	$F_{ST}sel>F_{ST}neu$ at low divergence times and $F_{ST}sel<F_{ST}neu$ at high divergence times, if $s_{1}\neq s_{2}$ and/or $t_{1}\neq t_{2}$.
Local selection (Charlesworth *et al.* 1997)	1	1−hs	1−s	1−s	1−hs	1	Prediction: $F_{ST}sel>F_{ST}neu$
	MM	Mm	mm	MM	Mm	mm	
Frequency-dependent (Charlesworth *et al.* 1997)	2(1−p)	1.5−p	1	2(1−p)	1.5−p	1	Prediction: $F_{ST}sel<F_{ST}neu$

Using simulations, Charlesworth *et al.* (1997) showed that local selection increases population differentiation relative to neutrality, while frequency-dependent selection decreases population differentiation. These results are somewhat intuitive given that in the first case genotype fitness differ among populations, but they are the same in the second case (Table 1). We do not expect to see selected sites switching from more to less differentiated than neutral ones (or vice versa) as a function of divergence time in neither of those scenarios. However, such a switch was observed in the case of divergent overdominance, with selected SNPs shifting from greater to lower divergence with respect to neutral sites.

To sum up, other forms of balancing selection can generate lower F${}_{\text{ST}}$ relative to neutrally evolving sites, as we observed at the HLA genes over long periods of time, but only divergent overdominance can also account for the additional feature of increased differentiation among recently diverged populations. Another non-exclusive mechanism that could explain increased differentiation at recently diverged populations, as well as decreased differentiation at longer divergence times, are shifts from balancing to positive selection, which have been reported in other genes (de Filippo *et al.* 2016).

Another dimension to be explored is the biological level of organization on which the selection regimes described in Table 1 can act on HLA genes (*e.g.*, SNPs, genes, groups of genes). Lighten *et al.* (2017) investigated differences in population differentiation on two levels of HLA variation: HLA alleles (which we refer to as “HLA haplotypes”), and HLA supertypes, which are broader categories that encompass groups of HLA haplotypes with similar peptide binding properties. Lighten *et al.* (2017) show that selection acts differently in those two levels, with HLA supertypes being stable over time and showing little differentiation, while the specific HLA alleles belonging to each supertype show extensive turnover (often with marked differences between populations).

We have touched on this issue by looking at one level of organization lower than Lighten *et al.* (2017), when we contrasted F${}_{\text{ST}}$ at HLA SNPs and haplotypes. We hypothesized that low population differentiation at HLA SNPs could be compatible with high differentiation at HLA haplotypes, relative to neutral sites. This would be expected if particular HLA proteins were locally selected by population-specific pathogens. However, our dataset did not have enough resolution to answer this question, and we found that population differentiation at HLA haplotypes is intermediate between HLA SNPs and neutral sites, and not significantly different from either. Although we did not find evidence for high differentiation at HLA haplotypes with the limited data we had access to, we believe this is an interesting open question that could be addressed when an appropriate dataset becomes available (one with phased HLA haplotypes in multiple populations, along with an extensive set of neutral markers to be used as control).

It is possible that selection acts simultaneously at different levels of organization at the HLA genes, and involves a combination of the regimes described in Table 1. It is important to acknowledge this possibility if we want to fully understand the processes maintaining genetic diversity at the HLA genes.

## Conclusion

We have explored the degree of population differentiation at HLA loci and compared it to that of neutrally evolving sites. Our findings confirm that tests for selection based on F${}_{\text{ST}}$ are sensitive to the frequency at which variants are segregating, an effect that is particularly pronounced for the HLA genes, which show highly skewed site frequency spectra (with an excess of intermediate frequency alleles). When this skew is accounted for, we find that over long spans of time HLA SNPs show decreased population differentiation.

We also found that for recent divergence (involving population pairs from the same continent) differentiation at SNPs in the HLA genes was often higher than for neutrally evolving sites. We used simulations to show that a regime of overdominant selection maintained constant for diverging populations cannot explain high differentiation at low divergence times. However, an alternative overdominance model where two divergent populations are under overdominance, but differ in the fitness values of the homozygotes (*i.e.*, divergent overdominance), reproduces the observed pattern of lower differentiation over long spans of time, and higher at shorter timescales.

A complete and systematic exploration of the different regimes of balancing selection and different levels of biological organization they act upon, along with their interactions with demographic history, represents an important challenge. Our results document that population differentiation can be informative in the process of proposing models and designing tests to understand the selective pressures acting on HLA loci.
